# Evaluation of Selected Oxidant/Antioxidant Parameters in Patients with Relapsing-Remitting Multiple Sclerosis Undergoing Disease-Modifying Therapies

**DOI:** 10.3390/antiox11122416

**Published:** 2022-12-07

**Authors:** Anna Bizoń, Justyna Chojdak-Łukasiewicz, Aleksandra Kołtuniuk, Sławomir Budrewicz, Anna Pokryszko-Dragan, Agnieszka Piwowar

**Affiliations:** 1 Department of Toxicology, Faculty of Pharmacy, Wroclaw Medical University, Borowska 211, 50-556 Wroclaw, Poland; 2 Department of Neurology, Wroclaw Medical University, Borowska 213, 50-556 Wroclaw, Poland; 3 Department of Nervous System Diseases, Faculty of Health Sciences, Wroclaw Medical University, Bartla 5, 51-618 Wroclaw, Poland

**Keywords:** multiple sclerosis, medicines, prooxidant–antioxidant balance

## Abstract

The aim of this study was to evaluate oxidative stress parameters, specifically the concentration of advanced oxidation protein products (AOPP) and ferric-reducing antioxidant power (FRAP), in the serum of patients with relapsing-remitting multiple sclerosis (RRMS). We also analyzed the relationships between each parameter and selected clinical/laboratory multiple-sclerosis-related parameters. The study group comprised 204 patients with RRMS and 29 healthy, age-matched controls. The concentration of AOPP was significantly higher in the RRMS patients than in controls. ROC analysis showed the ability of AOPP to distinguish between the patients with RRMS and controls (the value of AUC was 94.8%, with a sensitivity of 89.69% and specificity of 89.3%). AOPP and FRAP were significantly higher in male than in female RRMS patients. Correlations were found between AOPP and the laboratory markers of inflammation. AOPP differed in the subgroups of patients treated with particular medications. Our findings indicate an increase in the markers of oxidative stress in the serum of RRMS patients, possibly linked with chronic inflammation. Gender and type of treatment affected the markers of oxidative stress.

## 1. Introduction

Multiple sclerosis (MS) is a chronic, autoimmune, demyelinating disease of the central nervous system (CNS), with the onset most often in young people. MS can lead to physical disability, cognitive impairment and decreased quality of life [1]. The prevalence of MS constantly rises, especially in the developed countries of Western Europe and North America (>100 cases per 100,000 population) [2]. Currently, the rate of prevalence of MS is similar in Poland, ~110 per 100,000 population [3], and approximately 2000 new cases are diagnosed annually [4]. The most common type of MS (affecting about 85% of cases at onset) is relapsing-remitting multiple sclerosis (RRMS), which is characterized by periods of exacerbations (new or recurrent symptoms of neurological deficit) followed by a complete or partial recovery [5,6,7]. The core element of the RRMS background is an autoimmune response associated with an inflammatory cascade which results in a multifocal CNS injury. Current strategies for MS management include the treatment of acute relapses based on immunomodulatory or immunosuppressive modes of action aimed at the effective control of the disease activity, prevention of relapses and accumulating disability, as well as increasing the range of disease-modifying therapies (DMT) [8].

The complex pathogenesis of MS remains unclear. Interactions between genetic predispositions and environmental factors are supposed to initiate and modify dysregulated immune responses. Furthermore, immune-mediated inflammatory demyelination is accompanied by a slowly evolving neurodegenerative process leading to axonal loss. This component of the MS background is even less recognized and thus hardly addressed by therapeutic options [9]. 

There is some evidence that increased oxidative stress plays an important role in the pathogenesis of MS [10,11]. Oxidative stress is associated with DNA defects, abnormal gene expression, defective enzyme activities and energy failure [12,13]. Neurons and glial cells are particularly sensitive to these detrimental effects [14]. Mitochondrial dysfunction, which causes increased production of reactive oxygen species (ROS), seems to contribute to both the inflammatory and degenerative components of the MS background. A disturbed oxidant/antioxidant balance was demonstrated to enhance neuroinflammation (with reciprocal relationships), promote axonal injury and reduce myelin repair [15]. Furthermore, some relationships were shown between the markers of oxidative stress and functional/clinical measures of MS progression [16,17,18]. 

Investigation of oxidative stress with regard to the MS background may shed some new light upon the disease pathology, with possible diagnostic and therapeutic implications. Considering individual differences in MS course and response to treatment, there is an ongoing search for reliable biomarkers of MS activity and progression. Parameters of oxidative stress certainly deserve attention in this field, and thus they have already been investigated in MS patients [19,20]. One of these parameters is the concentration of advanced oxidation protein products (AOPP), a relevant factor in the pathogenesis of many diseases [21], including immune-mediated and inflammatory ones [20]. AOPP seem associated, i.a., with dendritic cell activation and play a crucial role in neuroinflammation and oxidation processes in neurodegenerative diseases [22,23]. AOPP in serum are related to gender (with significantly higher levels in males) and function of liver (where these products are primarily eliminated) [24]. The determination of ferric-reducing antioxidant power (FRAP) was used as a measure of antioxidant power [25].

The aim of this study was to evaluate oxidative stress parameters, specifically AOPP and FRAP in the serum of patients with RRMS. Another goal of this study was to analyze the relationships between these parameters and systemic measures of inflammation as well as disease-modifying therapies. 

## 2. Materials and Methods

### 2.1. Materials

Our study enrolled patients with RRMS who either were hospitalized or consulted with the Department of Neurology at Wroclaw Medical University between May and July of 2021. All the patients included had been diagnosed with RRMS according to McDonald’s criteria [26], were being treated with DMT, undergoing regular follow-up appointments and had fully documented courses of the disease. 

The following were exclusion criteria: (1) primary or secondary progressive types of the disease; (2) the occurrence of MS relapse within the preceding 3 months; (3) the initiation of or switching DMT within the preceding 6 months; (4) decompensated or uncontrolled systemic comorbidities; (5) addictions such as smoking or alcohol abuse. 

Finally, 204 patients were included (73 men and 131 women aged 24–69 years, with a median age of 43.0). The duration of disease and degree of disability (Expanded Disability Status Scale-EDSS) [27,28] were determined on the basis of medical records. DMT used in the study group included glatiramer acetate (GA) (*n* = 20), interferon beta (IFNβ-1a, IFNβ-1b, pegylated interferon beta) (*n* = 64), dimethyl fumarate (DMF) (*n* = 62), teriflunomide (TER) (*n* = 29) and fingolimod (FTY) (*n* = 29). Due to a small number of patients receiving natalizumab (6 patients) or ocrelizumab (3 patients), monoclonal antibody treatment was excluded from our study.

The control group consisted of 29 healthy, age-matched individuals (9 men and 20 women aged 25–68 years, with a median age of 41.0). 

Venous blood samples (approximately 15 mL) were collected from each participant according to standard procedures, after overnight fasting between 7 and 9 a.m. Samples were immediately centrifuged, and the serum was separated. The remaining serum was frozen in −80 °C, and oxidative stress parameters were assayed immediately after thawing. The total number of white blood cells and lymphocytes from a complete blood count and of C-reactive protein (CRP) were included into the investigation.

This study was approved by the ethics committee of Wroclaw Medical University, Poland (KBN No. 146/2022), and was conducted in accordance with the Helsinki Declaration. All participants provided written informed consent prior to their inclusion in this study.

### 2.2. Methods

#### 2.2.1. The Determination of AOPP in Serum

AOPP were assayed spectrophotometrically in 1 mL of diluted serum (10-fold in PBS) after an addition of 50 µL of 1.16 M potassium iodide (Cat. No.: BA3160117, POCH, Gliwice, Poland) and were dissolved in 100 µL of 10% acetic acid prepared from glacial acetic acid (Cat. No.: 64-19-7, Stanlab, Lublin, Poland) and 100 µM of chloramine-T (Cat. No.: 112256603, Chempur, Piekary Śląskie, Poland) as a substrate in accordance to a method described earlier [29]. After a short incubation time (15 s) at room temperature, a stop solution was added and the absorbance was read at λ = 340 nm using a visible spectrophotometer (SP-830 Plus, Metertech, Taipei, Taiwan). The unit was expressed as micromoles per liter (µM) of chloramine-T equivalent.

#### 2.2.2. The Determination of FRAP in Serum

FRAP was measured in diluted serum (10-fold in PBS) using the colorimetric method with ferric tripyridyltriazine described earlier [30]. The FRAP reagent was prepared by mixing 300 mM of acetate buffer, with a pH of 3.6, with 10 mM of 2,4,6-tripyridyl-s-triazine (TPTZ) (Cat. No.: Acros 168070050, Thermo Scientific, Vienna, Austria) and was dissolved in 40 mM of HCl and 20 mM of an aqueous solution of FeCl_3_ × 6H_2_O (Cat. No.: 119041804, Chempur, Poland). The reaction was started by adding 500 µL of the freshly prepared FRAP reagent to 100 µL of diluted serum. After 5 min of incubation at 25 ℃, the samples were centrifuged at 2000× *g* for 10 min. The absorbance was read at λ = 593 nm using a visible spectrophotometer (SP-830 Plus, Metertech, Taiwan). 

Using those methods, we compared the concentration of AOPP and the FRAP value between the patients with RRMS and controls, males, and females and the five subgroups of RRMS patients treated with particular DMT. Furthermore, the correlations between either AOPP or FRAP and selected clinical/laboratory parameters were investigated. 

### 2.3. Statistical Analysis

Statistical analysis was performed using the Statistica Software Package, version 13.3 (Polish version; StatSoft, Kraków, Poland). Values were expressed as the median, 1st quartile and 3rd quartile. Normality of the variables was tested using the Shapiro–Wilk test. Homogeneity of variance was assessed using Levene’s test. Differences between two groups were investigated using the non-parametric Mann–Whitney U test, while differences between the five subgroups were investigated using the Kruskal–Wallis one-way analysis of variance on ranks. Correlations were checked using Spearman’s rank correlation coefficient. In all instances, *p* < 0.05 was considered statistically significant.

## 3. Results

The basic characteristics of the patients with RRMS and the control group are presented in Table 1. The mean age did not differ between the groups. The patients with RRMS had a significantly higher concentration of AOPP (*p* < 0.0001) when compared to the control group, while the value of FRAP was similar in both groups (*p* = 0.2733).

**Figure 1:**
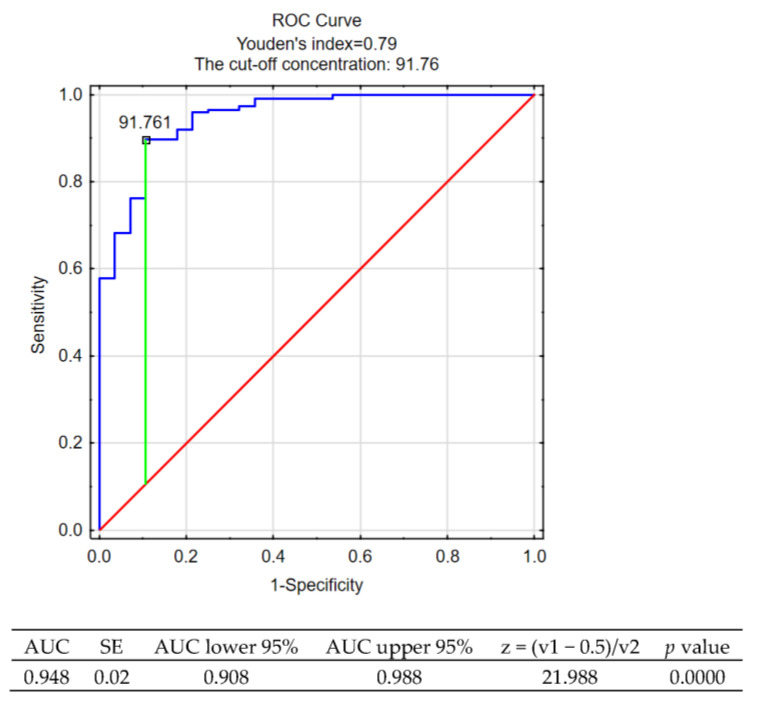
ROC curve of AOPP concentration for distinguishing patients with RRMS from the control group (x = 0.1071; y = 0.8969).

ROC analysis performed on the group of patients suffering from RRMS and on the control group indicated that a specified concentration of AOPP could be a powerful parameter to discriminate the patients with RRMS from healthy subjects. The cut-off concentration of AOPP was estimated as 91.761 [µM] (Figure 1). 

When we divided the patients with RRMS and the control group into gender subgroups, we found that in both groups the concentration of AOPP was significantly higher in male than in female subjects, while the value of FRAP was increased in male subjects when compared to female subjects. In the group of patients with RRMS, the median value of the disease duration and EDSS score were similar in the male and female subgroups (Table 2).

**Figure 2:**
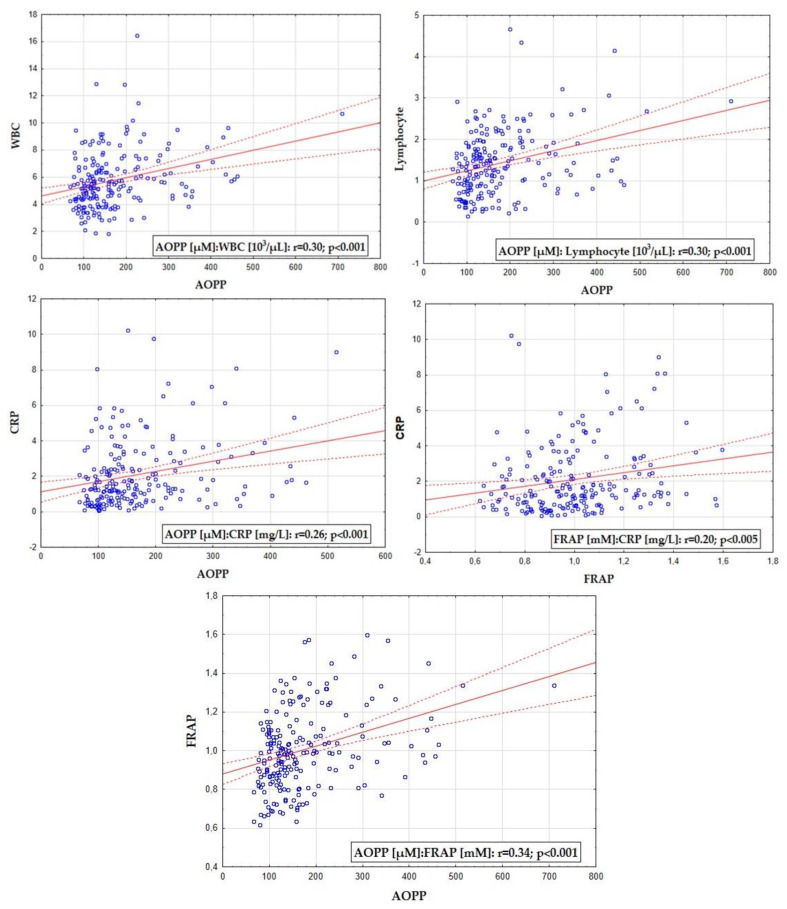
Statistically significant correlations between either FRAP or AOPP and WBC, lymphocytes, and CRP (for the whole study group). FRAP—ferric-reducing antioxidant power; AOPP—advanced oxidation protein products; CRP—C-reactive protein; WBC—white blood cells.

In the group of RRMS patients, the relationships were evaluated between either AOPP or FRAP and the other investigated parameters (Figure 2 and Table 3). The concentration of AOPP was significantly related to the number of WBC and lymphocytes. However, such a correlation was not found for the FRAP value. Furthermore, the value of FRAP and the concentration of AOPP showed a positive correlation with CRP in the whole study group and also in the female subgroup. 

All analyzed variables were compared between the subgroups of patients treated with particular DMT. There were statistically significant differences between these subgroups for disease duration, EDSS score and the WBC and lymphocyte count. Differences were also found in the concentration of AOPP, while the value of FRAP was similar across the subgroups (Table 4).

## 4. Discussion

An imbalance between the production and accumulation of oxygen reactive species was shown to contribute to MS-related demyelination and axonal damage within the CNS. Modulation of the prooxidant–antioxidant balance may represent one of the mechanisms involved in the effects of DMT [31,32]. Thus, measures of oxidative stress, analyzed in MS patients, might be useful in monitoring the processes underlying the disease pathology as well as in evaluating response to treatment. Among the oxidative stress parameters investigated in immune-mediated diseases, the concentration of AOPP seems to be the most relevant one. 

The second factor taken into consideration in this study was FRAP. FRAP is the only assay that measures antioxidants directly, when compared with other assays that measure the inhibition of free radicals, and can be used as a single test for the estimation of the total antioxidant capacity of blood [33]. Therefore, using both parameters, we could determine the changes in the prooxidant/antioxidant balance in the serum of patients with RRMS.

The determination of AOPP concentration was successfully used to confirm in vitro inflammatory status and dysregulated immune responses [34]. In the study by Obradovic et al. [20], the decrease in the concentration of AOPP was shown to be a good prognostic parameter for clinical outcomes in MS patients, including recovery after treatment of relapse [20]. A similar conclusion was made in the meta-analysis conducted by Rodrigues et al. [35], who claimed that AOPP may represent a new target for drug development in MS treatment and a possible biomarker to monitor the severity of the disease.

We found that the concentration of AOPP was about twofold higher in the RRMS patients than in controls, which is consistent with the study conducted by Obradovic et al. [20].

Similar results were also observed by other authors [36,37]. Furthermore, in the study conducted by Ljubisavljevic et al. [38], an elevation in AOPP concentration was revealed not only in plasma but also in the cerebrospinal fluid of patients with RRMS when compared to the control group. 

ROC analysis also showed the ability of AOPP to discriminate the RRMS patients from healthy controls. Thus, increased AOPP reflect enhanced oxidative stress in the course of the disease. 

The FRAP value did not differ between the patients with RRMS and controls. However, significant correlations regarding AOPP and FRAP in the study group suggest that increased oxidative stress could be balanced by antioxidant capacity. Findings from other studies in this field are inconsistent, reporting either a diminished value of the total antioxidant capacity (TAC) in MS patients [39] or no differences in total antioxidant status (TAS) between MS subjects and healthy controls [40]. 

Oxidative stress is supposed to contribute to both the inflammatory and degenerative component of the MS background. Analyzing the relationships between the oxidative stress measures and parameters of chronic inflammation in our study, we found that FRAP and AOPP correlated positively with CRP and that AOPP correlated likewise with WBC and lymphocyte count. 

Links between oxidative stress and inflammation were also suggested by the reports of other scientists [40,41].

However, it should be noted that the analyzed inflammatory parameters in the study group remained within normal limits. This is otherwise typical for MS because inflammatory activity takes place within the CNS and is not reflected in peripheral blood parameters. In addition, lowered lymphocyte count may occur in MS patients as a side effect of immunosuppressive DMT.

No significant relationships were found in the whole study group between either AOPP or FRAP and the clinical MS-related variables (disease duration or degree of disability in EDSS). Surprisingly, such correlations were found in the separately analyzed gender subgroups.

Thus, it is difficult to determine how the oxidative stress markers are associated with the progression/advancement of the disease.

It should be highlighted that due to the exclusion criteria (recent relapse and/or treatment failure), our RRMS patients presented a stable condition supposedly as a result of effective therapy. We made an attempt to compare the oxidative stress parameters in the subgroups receiving different DMT.

The highest concentration of AOPP was observed in the patients treated with GA and IFNβ, while the lowest was observed in the subgroups on DMF. These differences may be attributed to the modes of action for particular DMT. GA is a tetrapeptide, so it may become an additional source of amino acids and thus increase the concentration of AOPP (a marker of protein oxidation). Furthermore, the immunomodulatory properties of GA (affecting T cell profiles and regulation of dendritic cells and macrophages) might contribute to an increase in oxidative stress [42,43]. IFNβ is a polypeptide which exerts a pleiotropic anti-inflammatory effect and modulates cellular autoimmune responses [44]. Aldabah et al. observed a higher concentration of malondialdehyde and a decreased total antioxidant status (TAS) in RRMS patients treated with IFNβ and attributed these findings both to the disease and effects of the treatment [45]. On the other hand, DMF effects include immunomodulation as well as neuroprotection [46]. There is evidence that due to cooperation with nuclear factor kappa-light-chain-enhancer of activated B cells (NF-κB), DMF not only inhibits proinflammatory cytokine activity but also reduces oxidative stress and prevents emerging neuronal injury [47,48]. The FRAP value, unlike AOPP concentration, did not differ across these subgroups, which suggests that antioxidant capacity is not straightforwardly related to the modes of action of DMT.

However, these findings should be interpreted cautiously. It has to be considered that the subgroups on particular DMT significantly differed in number of patients, MS-related variables (disease duration and EDSS) and, presumably, also in the history and duration of treatment. Further studies, comprising more homogenous groups of patients and multidimensional analyses of the relevant variables, are necessary to investigate the relationships between oxidative stress parameters and treatment response in MS patients. 

The oxidative stress parameters in our study were not dependent on age but were significantly related to gender. Gender differences in MS patients are well known, with a greater prevalence but more favorable disease course in women [49,50]. However, these differences were usually attributed to the profile and activity of autoimmune responses, while their links with pro/antioxidative balance are less recognized. Sex hormones can enhance oxidative stress, which has been observed in patients with neurodegenerative disorders [51]. The study conducted by Dimitrijević et al. [52] on rats with experimental autoimmune encephalitis showed that a higher AOPP concentration in the spinal cord, which is linked with more severe neurological deficits, was revealed only in male, but not female, rats. Moreover, Stojić-Vukanić et al. [53] revealed sex differences in the response to treatment of experimental autoimmune encephalomyelitis with DMF. In our study, the comparison of the gender subgroups showed higher values of AOPP and FRAP in male RRMS subjects. Another difference concerned AOPP correlations with the inflammatory parameters, which were more significant in female patients. In addition, we tried to analyze gender differences in each of the DMT subgroups, but the sample sizes obtained were small and supposedly biased the results.

Overall, our study demonstrated an increased concentration of AOPP, as a marker of oxidative stress, in the serum of patients with RRMS. No relevant findings were observed for FRAP as a measure of antioxidant capacity. The relationships between AOPP and the systemic inflammatory parameters in the study group suggest that oxidative stress is linked to chronic inflammation. AOPP concentration was also affected by gender and particular disease-modifying treatments. Further investigations are necessary to determine the value of AOPP as a potential marker of disease progression or therapeutic response in MS.

## Figures and Tables

**Table 1:** Basic characteristics of patients with RRSM and control group.

Variable	Control Group	Patients with RRMS
Number of subjects	*n* = 29	*n* = 204
Sex [men/women]	9/20	73/131
Age [years]	41.0 (36.0–41.0)	43.0 (37.0–51.0)
Duration of RRMS [years]	N/A	12.0 (8.0–15.0)
EDSS	N/A	2.5 (1.5–3.5)
FRAP [mM]	0.9 (0.9–1.1)	1.0 (0.9–1.1)
AOPP [µM]	70.9 (46.7–79.7)	141.4 (109.4–201.2) *

Values shown were for the median, 1st quartile and 3rd quartile; * *p* < 0.0001 when compared to the control group. RRMS—relapsing-remitting multiple sclerosis; EDSS—expanded disability status scale; FRAP—ferric-reducing antioxidant power; AOPP—advanced oxidation protein products; N/A—not applicable.

**Table 2:** Characteristics of group of patients with RRSM and the control group divided according to gender.

Variable	Control Group		Patients with RRMS	
	Women	Men	Women	Men
Number of subjects	*n* = 20	*n* = 9	*n* = 131	*n* = 73
Age	41.0 (33.0–49.0)	41.0 (36.0–47.5)	43.0 (37.0–51.0)	42.5 (35.5–51.0)
Disease duration [years]	N/A	N/A	12.0 (8.0–15.0)	12.0 (8.5–16.0)
EDSS	N/A	N/A	2.5 (1.5–3.5)	2.5 (1.5–3.0)
FRAP [mM]	0.9 (0.8–0.9)	1.1 (1.0–1.1) *	0.9 (0.8–1.0)	1.1 (1.0–1.3) *
AOPP [µM]	52.5 (44.0–172.9)	77.5 (70.6–88.2) **	128.6 (102.8–172.9)	163.8 (128.9–229.5) **

Values shown were for the median, 1st quartile and 3rd quartile; */** *p* < 0.01 when compared to the female group. RRMS—relapsing-remitting multiple sclerosis; EDSS—expanded disability status scale; FRAP—ferric-reducing antioxidant power; AOPP—advanced oxidation protein products; N/A—not applicable.

**Table 3:** Correlations between either FRAP or AOPP and clinical/laboratory parameters (for gender subgroups).

Female Subgroup (*n* = 131)		
Age	NS	NS
Disease duration	NS	NS
EDSS	NS	r = 0.18; *p* < 0.041
WBC [10^3^/µL]	NS	r = 0.31; *p* < 0.002
Lymphocyte [10^3^/µL]	NS	r = 0.28; *p* < 0.001
CRP [mg/L]	NS	r = 0.31; *p* < 0.001
AOPP [µM]	r = 0.24; *p* < 0.001	-
**Male Subgroup (*n* = 73)**		
Age	NS	NS
Disease duration	NS	r = −0.30; *p* < 0.028
EDSS	NS	r = −0.27; *p* < 0.048
WBC [10^3^/µL]	NS	r = 0.29; *p* < 0.028
Lymphocyte [10^3^/µL]	NS	r = 0.31; *p* < 0.025
CRP [mg/L]	NS	NS
AOPP [µM]	NS	NS

RRMS—relapsing-remitting multiple sclerosis; FRAP—ferric-reducing antioxidant power; AOPP—advanced oxidation protein products; EDSS—expanded disability status scale; CRP—C-reactive protein; WBC—white blood cells; NS—not significant.

**Table 4:** Comparison of analyzed variables in the subgroups of RRMS patients treated with particular DMT.

Variable	Patients with RRMS				
	GA	IFNs	TER	FTY	DMF
Number of subjects	*n* = 20	*n* = 64	*n* = 29	*n* = 29	*n* = 62
Men/Women [number]	6/14	30/34	15/14	8/21	14/48
Age [years]	44.0 (36.0–53.0)	43.0 (39.0–51.5)	46.5 (41.5–57.0)	42.0 (35.0–48.0)	41.0 (35.0–47.0)
Disease duration [years]	10.5 (7.5–16.5)	12.0 (9.0–15.0)	13.0 (7.0–16.5)	14.0 (13.0–20.0)	8.0 (5.0–13.0) *
EDSS	2.0 (1.5–2.8)	2.0 (1.5–3.0)	3.0 (2.0–3.8)	4.0 (3.0–6.0)	2.0 (1.0–3.0) *
WBC [10^3^/µL]	6.7 (6.1–8.4)	5.4 (4.4–6.5)	5.6 (4.6–7.0)	4.6 (3.8–6.4)	5.1 (4.3–5.8) *
Lymphocytes [10^3^/µL]	1.9 (1.7–8.4)	1.5 (1.2–1.9)	1.5 (1.1–1.8)	0.5 (0.3–1.5)	1.0 (0.7–1.5) *
CRP [mg/L]	2.1 (1.1–3.7)	1.3 (0.7–2.1)	1.5 (0.9–2.4)	1.3 (0.6–2.5)	1.4 (0.7–3.0)
FRAP [mM]	1.0 (0.8–1.1)	1.0 (0.9–1.1)	0.9 (0.8–1.1)	1.0 (0.9–1.1)	1.0 (0.9–1.1)
AOPP [µM]	164.1 (122.6–242.1)	162.8 (120.7–221.9)	152.5 (112.2–218.2)	138.3 (109.4–172.9)	124.02 (97.4–157.8) *

Values shown were for the median, 1st quartile and 3rd quartile; * *p* < 0.01 when compared among five different treatment subgroups. RRMS—relapsing-remitting multiple sclerosis; GA—glatiramer acetate; IFNs—interferons; TER—teriflunomide; FTY—fingolimod; DMF—dimethyl fumarate; EDSS—expanded disability status scale; WBC—white blood cells; CRP—C-reactive protein; FRAP—ferric-reducing antioxidant power; AOPP—advanced oxidation protein products.

## Data Availability

All relevant data in the current study are available from the corresponding author on request.
